# Host Double Strand Break Repair Generates HIV-1 Strains Resistant to CRISPR/Cas9

**DOI:** 10.1038/srep29530

**Published:** 2016-07-12

**Authors:** Kristine E. Yoder, Ralf Bundschuh

**Affiliations:** 1 Department of Molecular Virology, Immunology and Medical Genetics, Center for Retrovirus Research, The Ohio State University Medical Center, Columbus, Ohio, USA; 2 Department of Physics, Department of Chemistry and Biochemistry, Division of Hematology, Department of Internal Medicine, Center for RNA Biology, The Ohio State University, Columbus, Ohio, USA

## Abstract

CRISPR/Cas9 genome editing has been proposed as a therapeutic treatment for HIV-1 infection. CRISPR/Cas9 induced double strand breaks (DSBs) targeted to the integrated viral genome have been shown to decrease production of progeny virus. Unfortunately HIV-1 evolves rapidly and may readily produce CRISPR/Cas9 resistant strains. Here we used next-generation sequencing to characterize HIV-1 strains that developed resistance to six different CRISPR/Cas9 guide RNAs (gRNAs). Reverse transcriptase (RT) derived base substitution mutations were commonly found at sites encoding unpaired bases of RNA stem-loop structures. In addition to RT mutations, insertion and/or deletion (indel) mutations were common. Indels localized to the CRISPR/Cas9 cleavage site were major contributors to CRISPR gRNA resistance. While most indels at non-coding regions were a single base pair, 3 base pair indels were observed when a coding region of HIV-1 was targeted. The DSB repair event may preserve the HIV-1 reading frame, while destroying CRISPR gRNA homology. HIV-1 may be successfully edited by CRISPR/Cas9, but the virus remains competent for replication and resistant to further CRISPR/Cas9 targeting at that site. These observations strongly suggest that host DSB repair at CRISPR/Cas9 cleavage sites is a novel and important pathway that may contribute to HIV-1 therapeutic resistance.

The major barrier to a cure for HIV-1 infection is the persistence of cells with an intact integrated provirus. This reservoir of latently infected cells is estimated to persist for decades^1^. An intriguing new strategy to eradicate cells harboring latent proviruses or to prevent HIV-1 replication utilizes the clustered regularly interspersed short palindromic repeat (CRISPR) genome editing technology^2^,^3^,^4^,^5^. The CRISPR system is derived from bacterial immune systems and is able to target double strand breaks at sequence specific sites^6^,^7^. The Cas9 DNA endonuclease is directed by a short guide RNA (gRNA) comprised of 20 bases of homology to a target DNA site. The *S. pyogenes* Cas9 protein also requires a proto-spacer adjacent motif (PAM) of sequence NGG in the target DNA immediately adjacent to the gRNA homology region. Cas9 induces a blunt end double strand break (DSB) 3 bp 5′ of the PAM.

The pathway of DSB repair in mammalian cells depends largely on the cell cycle^8^. During S and G_2_ phases of the cell cycle, repair may be performed by error-free homologous recombination. However, in resting cells DSBs are most commonly repaired by the error-prone non-homologous end joining pathway (NHEJ). This type of repair often produces insertions or deletions (indels) at the repair joint^9^. The error-prone nature of NHEJ has been useful for cellular genome editing by altering the sequence of genes to prevent their expression^10^,^11^. One proposed strategy includes targeting the long terminal repeats (LTRs) at both ends of the provirus. CRISPR DSBs in both LTRs would functionally delete the viral genome^2^,^3^,^12^. Alternatively, DSBs repaired by NHEJ at sensitive sites could prevent expression of the provirus or alter the reading frame of viral genes^4^,^5^.

HIV-1 readily develops resistance to anti-retroviral drugs^13^. HIV-1 viral evolution may result from errors introduced by reverse transcriptase (RT), RNA polymerase II, or APOBEC3G^14^,^15^. Evolution of the HIV-1 sequence in patients requires viral replication, but the integrated latent provirus sequence is not evolving^16^,^17^,^18^. The static HIV-1 proviral sequence makes the latent reservoir an attractive target for genome editing strategies.

We targeted CRISPR/Cas9 to the HIV-1 DNA genome at sites necessary for efficient viral replication and sequenced resistant strains^6^,^7^. *S. pyogenes* Cas9 has been shown to tolerate a variable number of mismatches between the gRNA and target DNA^7^,^19^. Because the viruses in these experiments were replicating, it was predicted that most resistance strains would display RT derived base substitutions within the gRNA targeted site. While RT mutations did lead to HIV-1 strains resistant to CRISPR editing, many of the resistant strains were due to DSB repair associated mutagenesis. These results suggest that CRISPR editing of the HIV-1 genome may yield proviruses that remain competent for replication.

## Results

### Human CD4+ T cell lines stably expressing Cas9 and gRNAs display reduced HIV-1 replication kinetics

We designed CRISPR gRNAs to target regions of the HIV-1 genome necessary for efficient viral replication (Supplementary Fig. S1). Targets included sequences adjacent to the viral TATA box, the trans-activation response (TAR) element, and the Rev response element (RRE). Alignment with the Los Alamos National Laboratory (LANL) database of HIV-1 subtype B isolates showed minimal sequence heterogeneity at each site (Supplementary Fig. S1). The TATA box is required for all viral transcription^20^. Although the TATA box is a conserved motif for RNA polymerase II transcription, the targeted HIV-1 sequences flanking the viral TATA box are not similar to human sequences (Supplementary Tables 1 and 2). Binding of HIV-1 Tat protein to the TAR RNA stem-loop is necessary for efficient viral transcription^21^,^22^. Finally, binding of the HIV-1 protein Rev to the RRE RNA stem-loop is required for efficient nuclear export of singly spliced and unspliced viral mRNAs^23^. The gRNA-RRE/*env* targets a region encoding both the RNA stem-loop RRE and the *env* gene.

**Figure 1:**
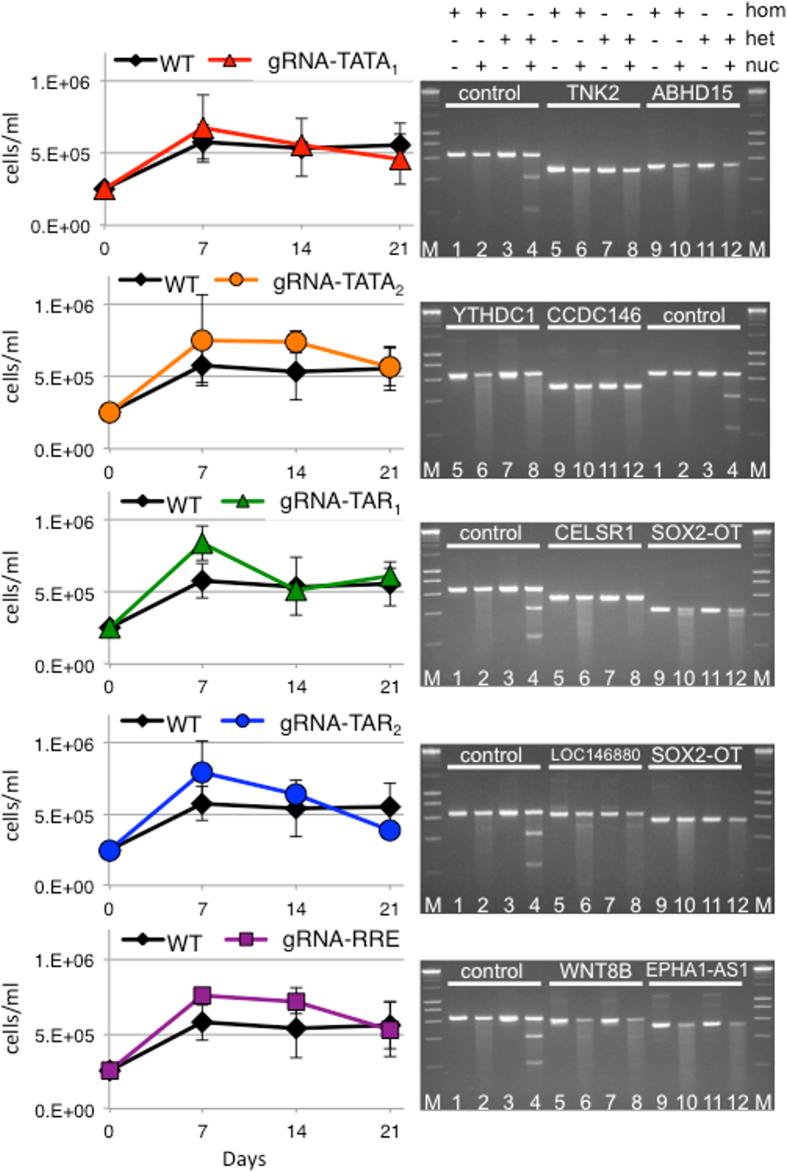
CRISPR expression does not impair cell growth or lead to significant off-target editing. (*Left*) Wild type SupT1 cells and CRISPR cell lines were plated at the same density. Viable cells were counted by trypan blue exclusion. The growth kinetics of the CRISPR expressing cells are similar to wild type cells. (*Right*) Surveyor assay of off-target editing at the two highest predicted sites in human genes. Positive and negative assay controls were included for each set of reactions. M, marker. The matrix indicates the inclusion of homoduplex DNA (hom), heteroduplex DNA (het), or Surveyor nuclease (nuc) for all the gels below. Lanes with Surveyor assay controls are indicated (control) and off-target sites are indicated by the name of the site in white text. Lanes 1 and 2, Surveyor negative control homoduplex shows background cleavage activity as a faint smear. Lanes 3 and 4, Surveyor positive control heteroduplex shows cleavage products as two faster mobility bands. Lanes 5 and 6, off-target 1 PCR products from wild type cells. Lanes 7 and 8, off-target 1 PCR products from wild type cells annealed to PCR products from CRISPR cells. Lanes 9 and 10, off-target 2 PCR products from wild type cells. Lanes 11 and 12, off-target 2 PCR products from wild type cells annealed to PCR products from CRISPR cells. The absence of faster mobility products in lanes 8 compared to lanes 6 and lanes 12 compared to lanes 10 indicate that off-target editing is minimal in these cells. CRISPR gRNA-TATA_1_ off-targets are TNK2 and ABHD15. gRNA-TATA_2_ off-targets are YTHDC1 and CCDC146. gRNA-TAR_1_ off-targets are CELSR1 and SOX2-OT. gRNA-TAR_2_ off-targets are LOC146880 and SOX2-OT. gRNA-RRE/*env* off-targets are WNT8B and EPHA1-AS1.

The human CD4+ T cell line SupT1 was stably transduced and selected to express the *S. pyogenes* CRISPR nuclease Cas9 and each gRNA^7^. Cellular growth kinetics were unaffected by CRISPR expression (Fig. 1). Because Cas9 may tolerate some mismatches between the gRNA and the DNA target, off-target digestion of cellular genes was also tested. The HIV-1 gRNAs in this study were selected based on the probability of off-target editing in the human genome *in silico* using two criteria: 1. the gRNAs have at least three mismatches to any human gene, and 2. the gRNAs have at least one mismatch in the 15 bases adjacent to the PAM^7^,^19^. A single exception was that gRNA-TAR_1_ has no mismatches in the 15 bases adjacent to the PAM compared to the off-target human gene CELSR1, a cadherin expressed in early embryogenesis (Supplementary Table 3). However, there are 4 total mismatches between gRNA-TAR_1_ and CELSR1. The two off target sites within human genes with the highest predicted probability for each gRNA were PCR amplified from wild type SupT1 and derivatives expressing Cas9 and the corresponding gRNA. The PCR products were analyzed by the Surveyor nuclease assay (Fig. 1). The two off-target sites tested for each gRNA did not appear to be significantly edited.

**Figure 2:**
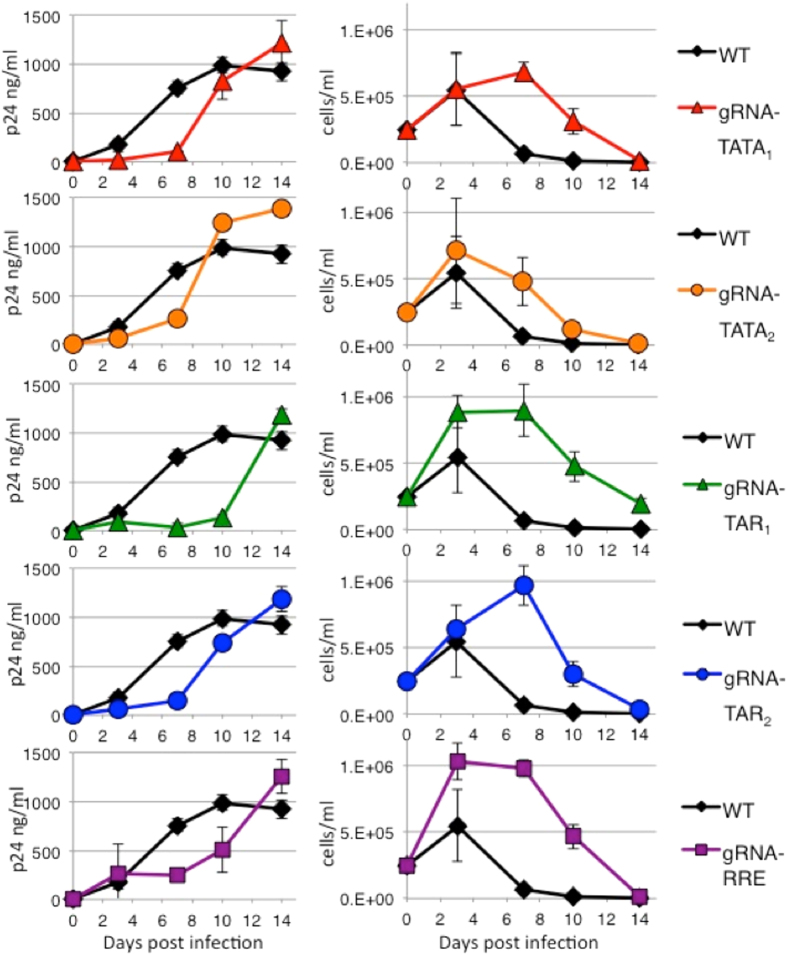
HIV-1 strain NL4-3 replication is delayed in human CD4+ T cell lines expressing CRISPR. Wild type human CD4+ T cell line SupT1 and derivatives expressing Cas9 and multiple gRNAs targeting HIV-1 were infected with strain NL4-3. (*Left*) Culture supernatants were assayed for the release of p24 as a measure of viral replication. All gRNAs suppress viral replication compared to wild type cells with no gRNA (WT). (*Right*) Cell viability was measured by trypan blue exclusion. As viral replication increases, cellular viability decreases. Error bars indicate the standard deviation between three separate infections.

**Figure 3:**
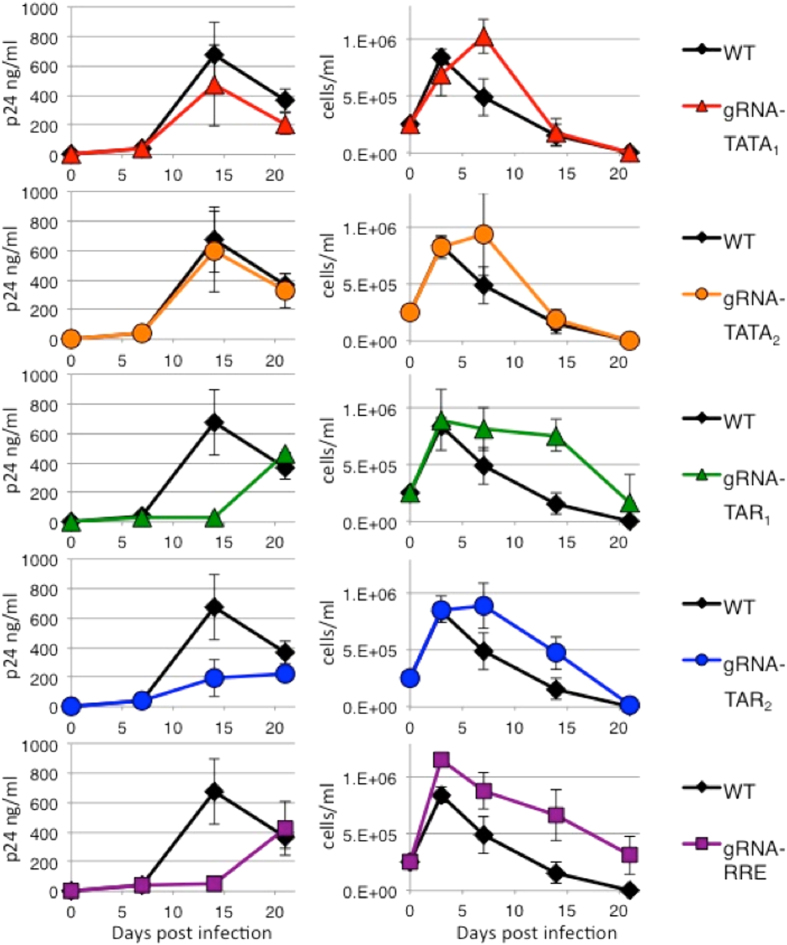
HIV-1 strain R7 replication in CRISPR human CD4+ T cell lines. Wild type human CD4+ T cell line SupT1 and derivatives expressing Cas9 and multiple gRNAs targeting HIV-1 were infected with strain R7. Compared to NL4-3, R7 is known to have slower growth kinetics. (*Left*) Culture supernatants were assayed for the release of p24 as a measure of viral replication. (*Right*) Cell viability was measured by trypan blue exclusion. As viral replication increases, cellular viability decreases. Error bars indicate the standard deviation between three separate infections. HIV-1 R7 infection of the gRNA-TATA_1_ cell line was included as a negative control. The R7 strain encodes one mismatch from gRNA-TATA_1_ 7 bp distal to the PAM. While Cas9 has been shown to tolerate limited mismatches for some gRNAs, empirically this single mismatch appears sufficient to prevent CRISPR/Cas9 gRNA-TATA_1_ editing and viral replication was equal to wild type cells.

SupT1 wild type and CRISPR cell lines were infected with HIV-1 strains NL4-3 (Fig. 2) and R7 (Fig. 3), which have different replication kinetics^24^. Viral replication was measured by the release of p24 capsid protein to the culture supernatants. HIV-1 is cytotoxic and the viability of the infected cells was measured by trypan blue exclusion. Increases of p24 correlated with decreases in cellular viability. In CRISPR gRNA-TATA_1_, gRNA-TATA_2_, and gRNA-TAR_2_ cells, the amount of NL4-3 p24 at 10 days post infection (dpi) is equivalent to wild type cells indicating the development of resistant strains (Fig. 2). CRISPR gRNA-TAR_1_ and gRNA-RRE/*env* cultures developed NL4-3 derived resistant strains by 14 dpi. Interestingly, the gRNAs targeting regions encoding RNA stem-loop structures suppressed viral replication longer than gRNAs targeting regions not predicted to form RNA secondary structures.

Replication of a second HIV-1 strain R7 was also evaluated in the CRISPR cell lines (Fig. 3). The R7 strain has a single base difference from NL4-3 in the region targeted by gRNA-TATA_1_. While Cas9 has been reported to tolerate some mutations between the gRNA and target DNA, this single base change allowed R7 to replicate in gRNA-TATA_1_ cells as efficiently as in wild type cells. In this case, a single base change was sufficient to prevent CRISPR editing and suppression of HIV-1 replication. The gRNA-TATA_2_ did not effectively inhibit R7 replication, similar to the poor inhibition of strain NL4-3. For both HIV-1 strains, gRNAs targeting regions encoding RNA stem-loop structures were better able to delay HIV-1 replication.

### HIV-1 strains resistant to CRISPR have mutations in the gRNA targeted site

**Figure 4:**
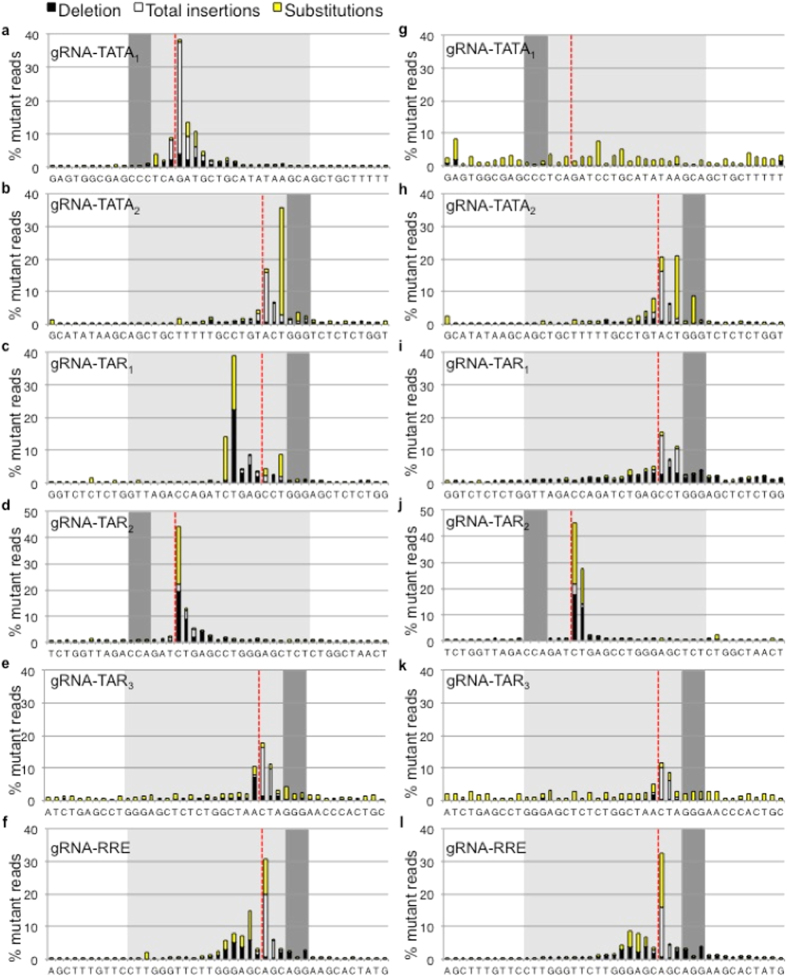
CRISPR resistant strains have mutations within the gRNA targeted site. The regions surrounding the CRISPR targeted sites were sequenced 5 weeks after infection. A window of 43 bp is shown and includes 20 bp of gRNA homology (light gray box), 3 bp PAM (dark gray box), and 10 bp of flanking sequence on each side. Red dotted lines indicate the point of cleavage by Cas9. Deletions, total insertions, and base substitutions are shown. The total number of mutations in the 43 bp window was set to 100% and the relative contribution of each mutation at each base is indicated as % mutant reads. The frequencies of mutations at flanking sites for all gRNAs are <3%. (**a–f**) NL4-3 derived CRISPR resistant strains. gRNA-TATA_1_ n = 1,473,434. gRNA-TATA_2_ n = 402,382. gRNA-TAR_1_ n = 655,747. gRNA-TAR_2_ n = 356,623. gRNA-TAR_3_ n = 62,704. gRNA-RRE/*env* n = 908,617. (**g–l**) R7 derived CRISPR resistant strains. gRNA-TATA_1_ n = 25,379. gRNA-TATA_2_ n = 149,465. gRNA-TAR_1_ n = 207,067. gRNA-TAR_2_ n = 279,998. gRNA-TAR_3_ n = 30,232. gRNA-RRE/*env* n = 749,407.

HIV-1 infections of CRISPR cell lines were continued for 5 weeks. Genomic DNA was isolated and regions surrounding the gRNA target sites were amplified and sequenced. Next-generation sequencing results were aligned to the reference genomes and analyzed for base substitutions as well as indels at each position (Fig. 4)^25^. Analysis of a 43 bp window, including the 20 bp of gRNA homology, the 3 bp PAM, and 10 bp of flanking sequence on either side of the targeted site, reveals that sequences flanking the gRNA targets displayed base substitution frequencies <3% and no indels. Mutation frequencies >3% were limited to the gRNA targeted region, including the Cas9 recognition PAM motif, suggesting that resistance mutations were specific to the CRISPR gRNA target.

### Indel mutations occur at the site of the Cas9 double strand break

**Figure 5:**
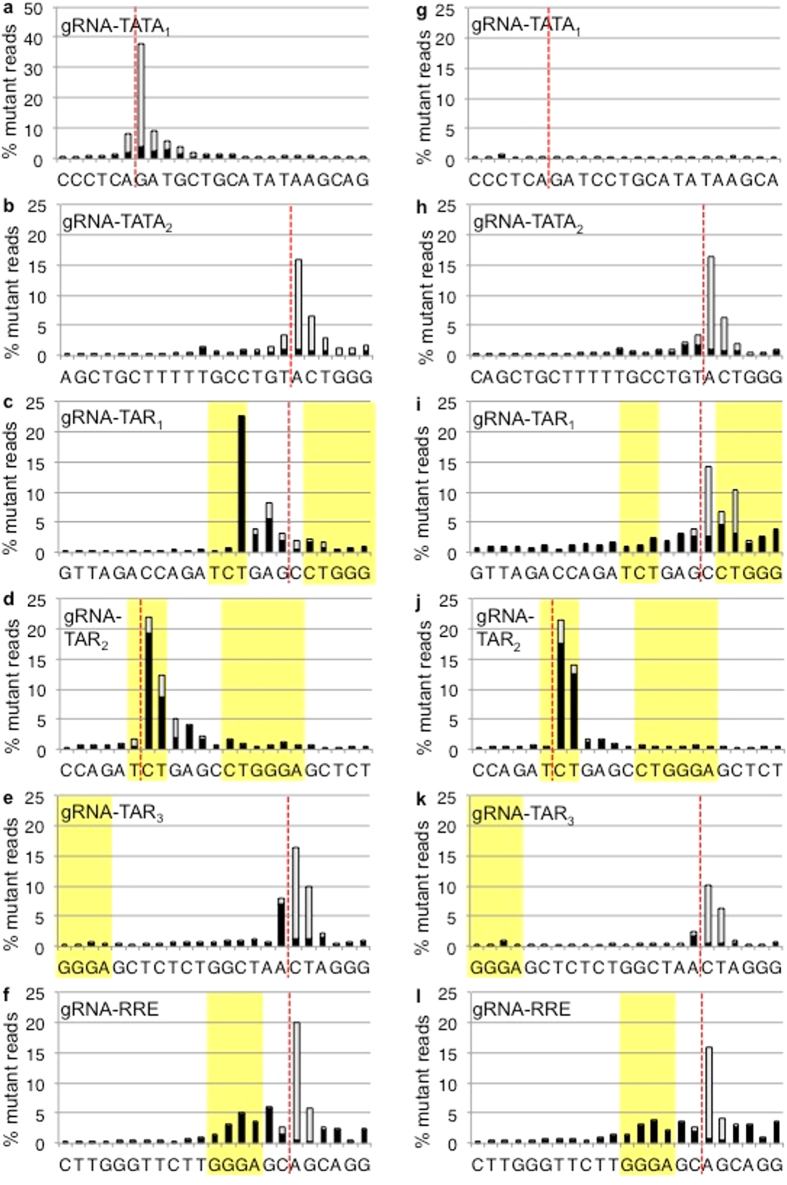
CRISPR resistant strains have indel mutations at the double strand break. Human CD4+ T cell lines expressing Cas9 and multiple gRNAs were infected with (**a–f**) the strain NL4-3 or (**g–l**) the strain R7. CRISPR resistant strains were characterized for indel mutations 5 weeks after infection. All resistant strains display indels. R7 infection of gRNA-TATA_1_ cells is a negative control and displays the background level of indels. The 23 bp gRNA targeted sequence is shown. Red dotted lines indicate the site of Cas9 cleavage. Black bars indicate a deleted base. Light gray bars indicate insertions. Yellow boxes highlight bases that encode a bulge or loop in an RNA structure.

Indels were common precisely at the CRISPR/Cas9 induced DSB for all the gRNAs with both viral strains, excluding R7 infection of naturally resistant gRNA-TATA_1_ cells (Fig. 5). CRISPR resistant strains arising from NL4-3 infection of the gRNA-TATA_1_ cell line showed the greatest bias of indels at the cleavage site (Fig. 5a). RT derived indels are infrequent and are associated with a run of at least 3 bases, which was not observed with CRISPR resistance indels at the Cas9 cleavage sites^14^. The frequency of indels associated with PCR and Illumina sequencing is <5% with even distribution throughout amplicons^15^. The exact locations at the Cas9 cleavage sites indicate that CRISPR resistance associated indels are not experimental artifacts. Single base insertions included all nucleotides with no apparent bias (Supplementary Fig. 2). This suggests error-prone host DSB repair leads to resistant strains.

**Figure 6:**
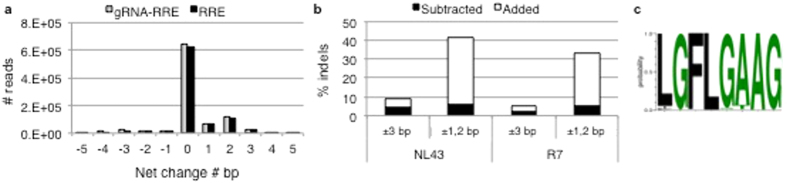
Resistance mutations associated with gRNA-RRE/*env* preserve the *env* reading frame. CRISPR gRNA-RRE/*env* targets the RRE and the *env* gene. (**a**) The net change in number of bases of the 23 bp gRNA or the 234 bp RRE sequence for all reads derived from NL4-3 infection. Most reads have no frame change, but net additions of 1 and 2 bp are present. (**b**) Indel reads were characterized for altered reading frame for both NL4-3 and R7 infections at the 23 bp gRNA sequence. The total number of bases either increased (Added) or decreased (Subtracted). In frame insertions or deletions were either +3 or −3 bases, respectively. Frameshifts were altered by 1 or 2 bases. (**c**) LANL protein sequence logo of the HIV-1 subtype B Env amino acids L520 to G527 encoded by the bases targeted by gRNA-RRE/*env*. The most common variant encodes NL4-3 Env A525, but 6% of patient samples encode NL4-3 Env T525.

The gRNA-RRE/*env* targets a region encoding the *env* gene and RRE RNA stem-loop. The gRNA-RRE/*env* resistant strains showed the highest frequency of multi-base insertions compared to our other gRNAs (Supplementary Fig. 2) and had deletions at several bases encoding the RRE bulge (Fig. 5f). These observations suggested that balanced indels possibly maintain the *env* reading frame. Defective proviral genomes were not filtered from the cultures or the sequence analysis, allowing detection of frameshifted *env*. Indels of 1 or 2 bp result in premature stop codons in the *env* gene in both NL4-3 and R7 strains. However, the majority of total reads display no net change in the number of bases (Fig. 6a). Less than half of the indel mutations appear to have changes of 1 or 2 bp (41% NL4-3, 33% R7) that are likely defective viruses (Fig. 6b). Although CRISPR/Cas9 induced DSBs are often repaired with only a single base insertion, a significant number of gRNA-RRE/*env* targeted sites were altered by ±3 bp preserving the *env* reading frame (8.7% NL4-3, 5.6% R7)^6^,^26^. These values remained constant when the entire RRE sequence was evaluated, suggesting no additional indels outside the gRNA targeted site (Fig. 6). While a significant minority of viral sequences were likely inactivated by frameshift of *env*, our data suggest that successful CRISPR/Cas9 editing within an HIV-1 gene often results in no net change of reading frame. Proviruses that retain the correct reading from following editing are more likely to be replication competent compared to proviruses with altered reading frames.

### Base substitution mutations are not limited to the Cas9 double strand break site

**Figure 7:**
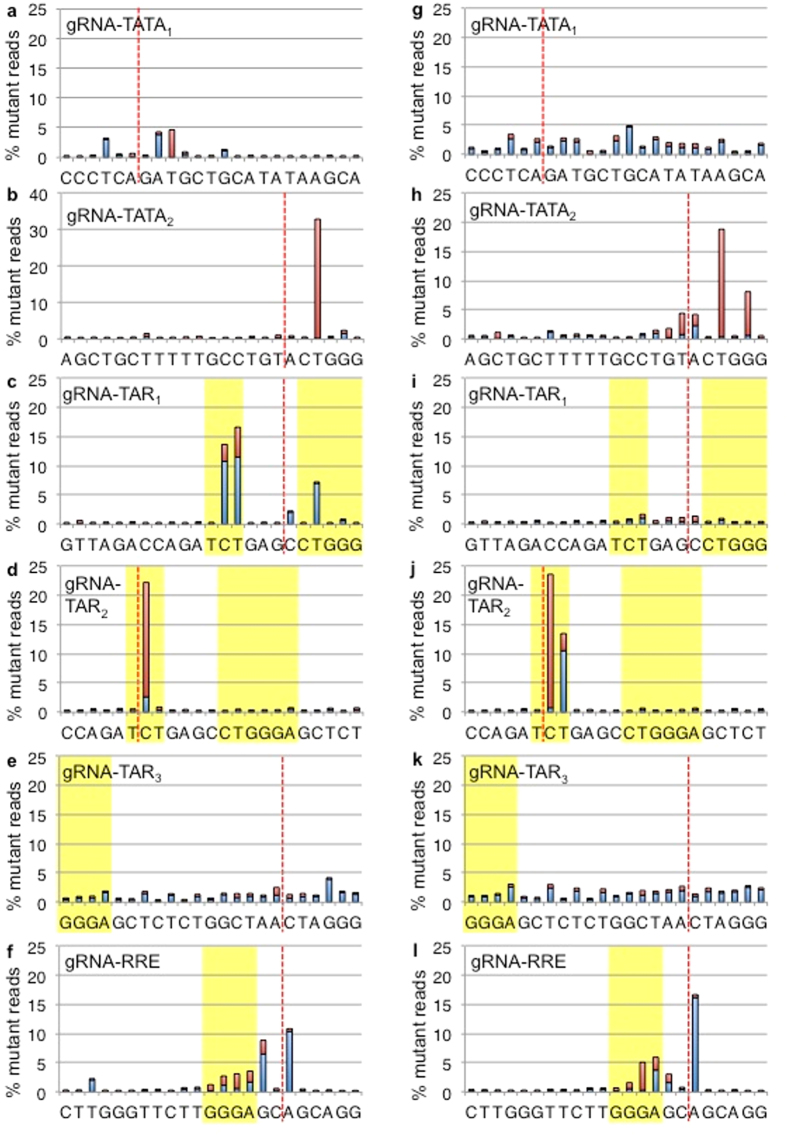
Base substitutions of CRISPR resistant strains present at variable positions of the gRNA homology site. CRISPR cell lines were infected with (**a–f**) strain NL4-3 or (**g–l**) strain R7. CRISPR resistant strains were characterized for transition (blue bars) and transversion (red bars) mutations. The 23 bp gRNA targeted sequence is shown. Red dotted lines indicate the site of Cas9 cleavage. Yellow boxes highlight bases that give rise to unpaired nucleotides of an RNA structure.

In addition to indel mutations, base substitutions were also observed in the resistant strains (Fig. 7). PCR and Illumina sequencing associated base substitutions are evenly distributed between transitions and transversions with transversions biased to C to A and G to T^15^. These characteristic mutations were not observed with the CRISPR resistance associated sequences, suggesting the observed base substitutions are not artifacts. HIV-1 RT favors transitions >3 fold over transversions *in vivo*^14^,^15^. R7 is naturally resistant to gRNA-TATA_1_ and viral sequences from this infection displayed bias to transitions, consistent with RT derived mutagenesis (Fig. 7g).

Strains with resistance to gRNA-TATA_2_ had a pronounced transversion of T to G immediately adjacent to the PAM (Fig. 7b,h). This base substitution likely occurred during reverse transcription at the first template switch. Although transversions are typically less common RT mutations, this T is the first nucleotide synthesized after the minus strand transfer and immediately follows a tract of three guanines; taken together, this is suggestive of an RT derived base substitution.

Base substitutions associated with gRNAs targeting sites encoding TAR and RRE frequently clustered at regions of unpaired bulges or loops in the RNA structures (Fig. 7c,d,f,j and l). The TAR RNA stem-loop has a UCU bulge encoded by TCT^22^. The HIV-1 protein Tat binds only to the first U of this unpaired bulge^27^. All of the unpaired bases of the TAR loop are bound by cyclin T1^28^. The first T encoding the UCU bulge and the bases encoding the TAR loop were never altered. However, mutations were readily observed for the remaining CT bases encoding the TAR bulge. Similarly indel and base substitution mutations occurred at bases encoding the bulge of the RRE (Figs 5f,l and 7f,l). The most frequently observed base substitutions at the RRE bulge region led to silent mutations NL4-3 and R7 Env(A525A) or R7 Env(G522G) (Fig. 6c). One base substitution mutation led to NL4-3 Env(A525T), which occurs in 6% of isolates in the LANL database (Fig. 6c). The base substitution mutations of gRNA-TAR_1_ and gRNA-RRE/*env* favor transitions, suggestive of RT derived mutations. These RT derived CRISPR resistance mutations appear to favor unpaired bases of RNA structures.

**Figure 8:**
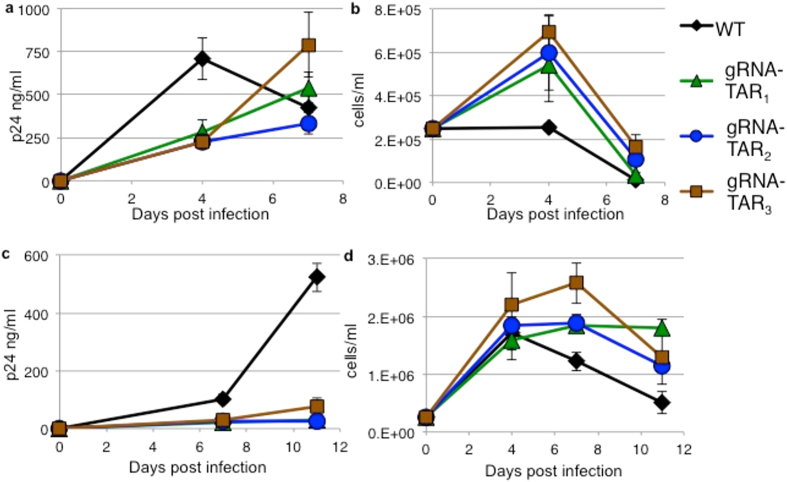
Three CRISPR gRNAs targeting TAR have similar effects on HIV-1 replication. Wild type human CD4+ T cell line SupT1 and derivatives expressing CRISPR Cas9 and three gRNAs targeting HIV-1 TAR were infected with (**a,b**) strain NL4-3 or (**c,d**) strain R7. Data from infection of four different cell lines are shown for direct comparison of the efficiencies of the TAR gRNAs. All three TAR gRNAs suppress HIV-1 replication equally. (**a,c**) HIV-1 replication was measured by the release of p24 to the culture supernatants. (**b,d**) Cellular viability was measured by trypan blue exclusion. Error bars indicate the standard deviation between three infections.

To test the relationship of resistance mutations and the unpaired regions of RNA stem-loops, a new CRISPR/Cas9 target in TAR was chosen to maximize base pairing, gRNA-TAR_3_ (Supplementary Fig. S1). Direct comparison of infection of the three TAR gRNA cell lines revealed similar inhibition of viral replication, indicating gRNA-TAR_3_ was not better at suppressing HIV-1 (Fig. 8). Similar to the other gRNA resistant strains, gRNA-TAR_3_ resistant strains displayed indels at the CRISPR/Cas9 cleavage site (Fig. 5e,k). However, the base substitution profile of gRNA-TAR_3_ shows no favored sites as seen with gRNA-TAR_2_ and appears more similar to the background base substitution profile seen with the negative control R7 infection of gRNA-TATA_1_ cells (Fig. 7e,k). Thus CRISPR/Cas9 targeting of sites encoding a fully base paired RNA structure and/or protein-binding sequence may be more prone to indel-mediated resistance.

The mutation profiles of NL4-3 and R7 infections were strikingly similar for all gRNAs except gRNA-TAR_1_. The resistance strains of the faster replicating NL4-3 virus were more likely to have mutations at the TAR bulge (Figs 5c and 7c) while R7 derived strains displayed indels at the DSB (Figs 5i and 7i). The mutation profiles are suggestive of NL4-3 derived resistant strains arising from reverse transcription and R7 derived resistant strains originating from DSB repair. While two distinct profiles were observed for gRNA-TAR_1_, the mutation profiles were similar among the other gRNAs tested. Although the genetic profiles of HIV-1 strains resistant to CRISPR gRNAs may display some variability, the majority of gRNAs may produce a single profile of resistance mutations.

## Discussion

Similar to other recent studies, our results demonstrate that both viral RT and host DSB repair pathways mediate resistance to CRISPR/Cas9 editing of HIV-1^29^,^30^. This is the first study to analyze CRISPR gRNA resistant strains arising from two strains of HIV-1 by next generation sequencing analysis. Replicating infections in CD4+ T cell lines allow for the identification of RT induced mutations. These were typically base substitutions within the gRNA homology region. In the case of CRISPR targets encoding RNA stem-loop structures, base substitutions with the signature of RT derived mutations occurred at bases encoding unpaired regions of the structure. Thus the bases that encode paired regions of RNA stem-loops are less likely to give rise to CRISPR resistant strains. However, results from a gRNA targeting a nearly fully base paired region of TAR show that mutations arise at the DSB, suggestive of repair mediated resistance.

Some studies suggest that CRISPR targeting of the HIV-1 LTRs may effectively delete the provirus by inducing DSBs at both repeats. The sequencing analysis here would not reveal such events. However, genome editing at multiple sites to generate large deletions may not be reasonably efficient^31^. In our experiments the strategy to prevent replication by deleting the provirus through targeting the LTRs was not efficient. Another recent study of HIV-1 strains resistant to a CRISPR gRNA also targeted the gRNA-TATA_1_ site^29^. A similarly generated CD4+ T cell line expressing the gRNA was infected with HIV-1 strain LAI and resistant strains displayed insertions and base substitutions.

Host DNA repair of the CRISPR/Cas9 induced DSBs appears to be a major pathway for HIV-1 to develop resistant strains. Cas9 has been reported to tolerate some mismatches and single base indels in the gRNA homology region^7^,^19^,^32^. The sequencing of resistant strains here suggested that among this small set of gRNAs, single base mismatches or indels were sufficient to confer resistance to CRISPR editing. The error-prone repair events generating a single base indel resulted in sequences no longer recognized by the gRNA. While this may be an acceptable outcome for genome editing of cellular genes, this may not be the case for disabling the HIV-1 provirus. Indels in noncoding regions, such as the LTRs, allow continued HIV-1 replication. While 1 and 2 bp indels in coding regions will effectively alter the reading frame of viral genes, we also observed indels altering the coding sequence by 3 bp, effectively preserving the reading frame. Viral sequences that maintain the reading frame were selected in these experiments, but would also undergo positive selection in patients.

CRISPR genome editing is an attractive method to incapacitate the HIV-1 provirus in latent reservoir cells since it is a static sequence^33^. The lack of reverse transcription in latent cells could suggest that viral resistance to CRISPR/Cas9 will not occur. However, our results indicate that HIV-1 CRISPR/Cas9 resistant strains arise by error-prone host DSB repair. Thus host DSB repair may result in a successfully CRISPR/Cas9 edited provirus that remains competent for replication. None of the observed DNA repair derived indels are present in the LANL database, suggesting that selective pressure from the CRISPR gRNAs leads to an unpredictable resistance pattern.

These results predict several features may be required for a successful CRISPR strategy targeting the HIV-1 provirus. A unique advantage to the CRISPR genome editing technology is the ability to multi-plex gRNAs. Targeting two gRNAs to delete relatively short regions of the HIV-1 genome may be more effective than attempting to delete the entire provirus. Targeting unstructured regions of the LTRs appears to quickly lead to resistance. Better strategies may include targeting multi-plexed gRNAs to RNA stem-loop structures or protein coding regions where single base indels will be more detrimental to the virus.

## Methods

### Cells

All cell culture reagents were from Life Technologies. Human CD4+ T cell line SupT1 was cultured in RPMI with 10% fetal bovine serum, penicillin, and streptomycin. Stable transductions of CRISPR genome editors were selected with 2 μg/ml puromycin. 293T cells for transfections were cultured in DMEM with 10% fetal bovine serum, penicillin, and streptomycin.

### Genome editors

CRISPR gRNAs were designed using the web tool cripsr.mit.edu^19^. The conservation of target sequences among HIV-1 patient samples was analyzed with the Los Alamos National Lab HIV sequence database QuickAlign v2 tool. Synthetic oligonucleotides (Supplementary Table 7; Integrated DNA Technologies) encoding the CRISPR gRNAs were subcloned to pLentiCRISPRv2 (Addgene) at BsmBI restriction sites^7^,^34^.

### Lentiviral vectors and HIV-1 strains

Human fibroblast 293T cells were transfected by calcium phosphate precipitation to generate HIV-based vector particles and HIV-1 virus. Lentiviral vector particles encoding CRISPR gRNAs and *Cas9* were generated by transfection with pVSV-G, p▵R9, and pLentiCRISPRv2. Although delivered by a lentiviral vector, *Cas9* and gRNA gene expression are not dependent on the vector encoded LTR or RRE. Human fibroblast 293T cells were transfected with plasmids encoding the HIV-1 proviruses pR7-GFP or NL4-3^35^,^36^. Culture supernatants were collected, filtered, and frozen at −80 °C.

### Genome editor cell lines

SupT1 cells were transduced with CRISPR vector particles at 0.1 MOI in the presence of 4 μg/ml DEAE dextran (Sigma Aldrich). Selection media containing puromycin was added to cells two days following transduction.

### Off-target editing analysis

Potential off target sites in coding regions of the human genome were identified by the web-based tool crispr.mit.edu (Supplemental Tables 1–6). Genome editing activity at off-target sites was measured by the Surveyor mutation detection kit (Integrated DNA Technologies). PCR primers were designed to amplify 400–600 bp regions around the two off target sites in coding regions with the least number of mismatches for each gRNA (Supplementary Table 8). Following transduction with the CRISPR lentiviral vector, puromycin selection, and continuous culture for 6 weeks, genomic DNA was purified (Qiagen DNeasy kit). Off target sites were amplified with the proof-reading enzyme Platinum Taq DNA Polymerase High Fidelity (Supplementary Table 7; Life Technologies). The products from off-target PCR reactions were gel purified, annealed as homologous or heterologous pairs, and digested with the Surveyor nuclease following the manufacturer’s instructions (Supplementary Table 8). Nuclease reactions were resolved with a 2% agarose gel stained with ethidium bromide and imaged with a Bio-Rad Gel Doc imaging system.

### HIV-1 infections

CRISPR expressing cell lines were infected with two strains of replicating HIV-1 virus, NL4-3 and R7. Strain R7 does not express the viral accessory gene *vpr*, *vpu*, or *nef* and is less cytotoxic than NL4-3^24^. NL4-3 does express all the viral accessory genes. Cells were infected with 125 ng p24 per 10^6^ cells. Culture supernatants were collected at multiple days post infection and assayed for viral replication by release of p24 into the media (ZeptoMetrix RETROtek HIV-1 p24 antigen ELISA, Fisher Scientific). Cells were also counted and assayed for viability by trypan blue exclusion (Sigma Aldrich).

### Resistance mutations

HIV-1 infections were continued for 21 days in 4 replicates per cell type and virus strain. The virus supernatant was passaged to uninfected CRISPR cells. Infection was visually confirmed by the presence of syncytia. Two weeks later the genomic DNA was purified (Qiagen DNeasy kit). A 504 bp amplicon of the LTR encompassing five gRNA target sites was amplifed with primers oKEY764 and oKEY782 with Platinum Taq High Fidelity (Life Technologies). A 501 bp region surrounding the target site of gRNA-RRE/*env* was amplified with primers oKEY217 and oKEY271. After PCR amplification, the products from replicate wells were pooled and paired-end sequenced by Illumina MiSeq PE300 v3 (Genomic Services Laboratory, HudsonAlpha Institute).

### Bioinformatics analysis

Sequences were aligned to the NL4-3 and R7 reference genomes. Base substitutions, insertions, and deletions were analyzed with a base quality cutoff of 0.001^25^. A 43 bp window with 10 bp flanking sequences on either side of the 23 bp gRNA target (20 bp gRNA homology and 3 bp PAM) was analyzed for mutations. The number of mutations at each base position is expressed relative to the total number of indels and base substitutions in the 43 bp window.

## Additional Information

**How to cite this article**: Yoder, K. E. and Bundschuh, R. Host Double Strand Break Repair Generates HIV-1 Strains Resistant to CRISPR/Cas9. *Sci. Rep.* **6**, 29530; doi: 10.1038/srep29530 (2016).

## Supplementary Material

Supplementary Tables 1 to 6

Supplementary Figures and Tables
