# Supplementary material for: Host Double Strand Break Repair Generates HIV-1 Strains Resistant to CRISPR/Cas9
Source: Sci Rep. 2016 Jul 12;6:29530. doi: 10.1038/srep29530 (PMC4941621; doi:10.1038/srep29530)
Supplement: Supplementary Figures and Tables [file srep29530-s2.pdf]

Host Double Strand Break Repair Generates HIV-1 Strains Resistant to CRISPR/Cas9

Kristine E. Yoder,[a]* and Ralf Bundschuh[b]

[a]Department of Molecular Virology, Immunology and Medical Genetics, Center for Retrovirus Research, The Ohio State University Medical Center, Columbus, Ohio, USA; [b]Department of Physics, Department of Chemistry and Biochemistry, Division of Hematology, Department of Internal Medicine, Center for RNA Biology, The Ohio State University, Columbus, Ohio, USA

*Address correspondence to Kristine E. Yoder, yoder.176@osu.edu.

Supplementary Figure 1

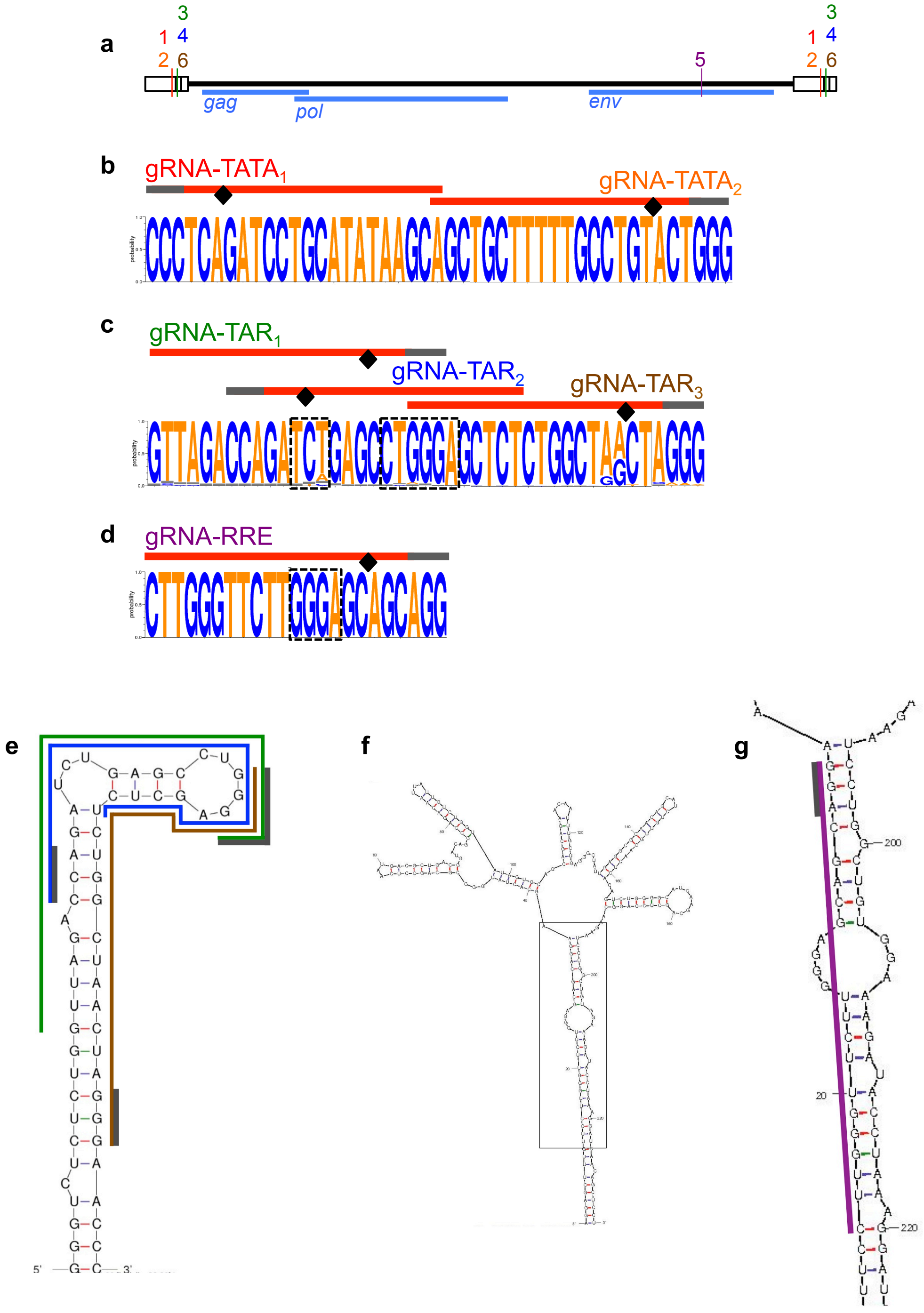

Supplementary Figure. 1. CRISPR gRNAs target conserved sequences in HIV-1 subtype B. (**a**) Cartoon of the HIV-1 genome indicating the long terminal repeats at each end as well as the *gag*, *pol*, and *env* genes. The relative locations of the gRNAs are indicated by colored lines and numbers as follows: gRNA-TATA$_1$ as red 1, gRNA-TATA$_2$ as orange 2, gRNA-TAR$_1$ as green 3, gRNA-TAR$_2$ as blue 4, gRNA-RRE/*env* as purple 5, and gRNA-TAR$_3$ as brown 6. (**b-d**) HIV-1 sequence logos indicate the relative sequence conservation at each base position in subtype B isolates curated at the Los Alamos National Lab HIV database. Red lines indicate the gRNA 20 bp homology region. Grey lines indicate the 3 bp PAM signal. Black diamonds indicate the site of Cas9 cleavage. (**b**) gRNA-TATA$_1$ and gRNA-TATA$_2$ are shown relative to each other and the HIV-1 sequence. The gRNA-TATA$_1$ targets a DSB 5' of the HIV-1 TATA box. The gRNA-TATA$_2$ targets the sequence between the HIV-1 TATA box and the TAR element. (**c**) gRNA-TAR$_1$, gRNA-TAR$_2$, and gRNA-TAR$_3$ partially overlap each other as well as bases encoding the TAR RNA bulge and loop. Dashed black boxes indicate bases that are unpaired in the TAR RNA stem-loop encoded by this region. (**d**) The gRNA-RRE/*env* is shown with HIV-1 subtype B sequence logo. Dashed black box indicates bases that are unpaired in the RRE RNA stem-loop encoded by this region. (**e**) The TAR RNA stem loop secondary structure is shown with gRNA binding sites shown as: gRNA-TAR$_1$ in green, gRNA-TAR$_2$ in blue, gRNA-TAR$_3$ in brown. PAM sequences for each gRNA are indicated by gray lines. (**f**) The RRE RNA stem loop secondary structure is shown. The boxed region is shown in (**g**) with the gRNA-RRE/*env* binding site shown in purple with the PAM sequence in gray.

Supplementary Figure 2

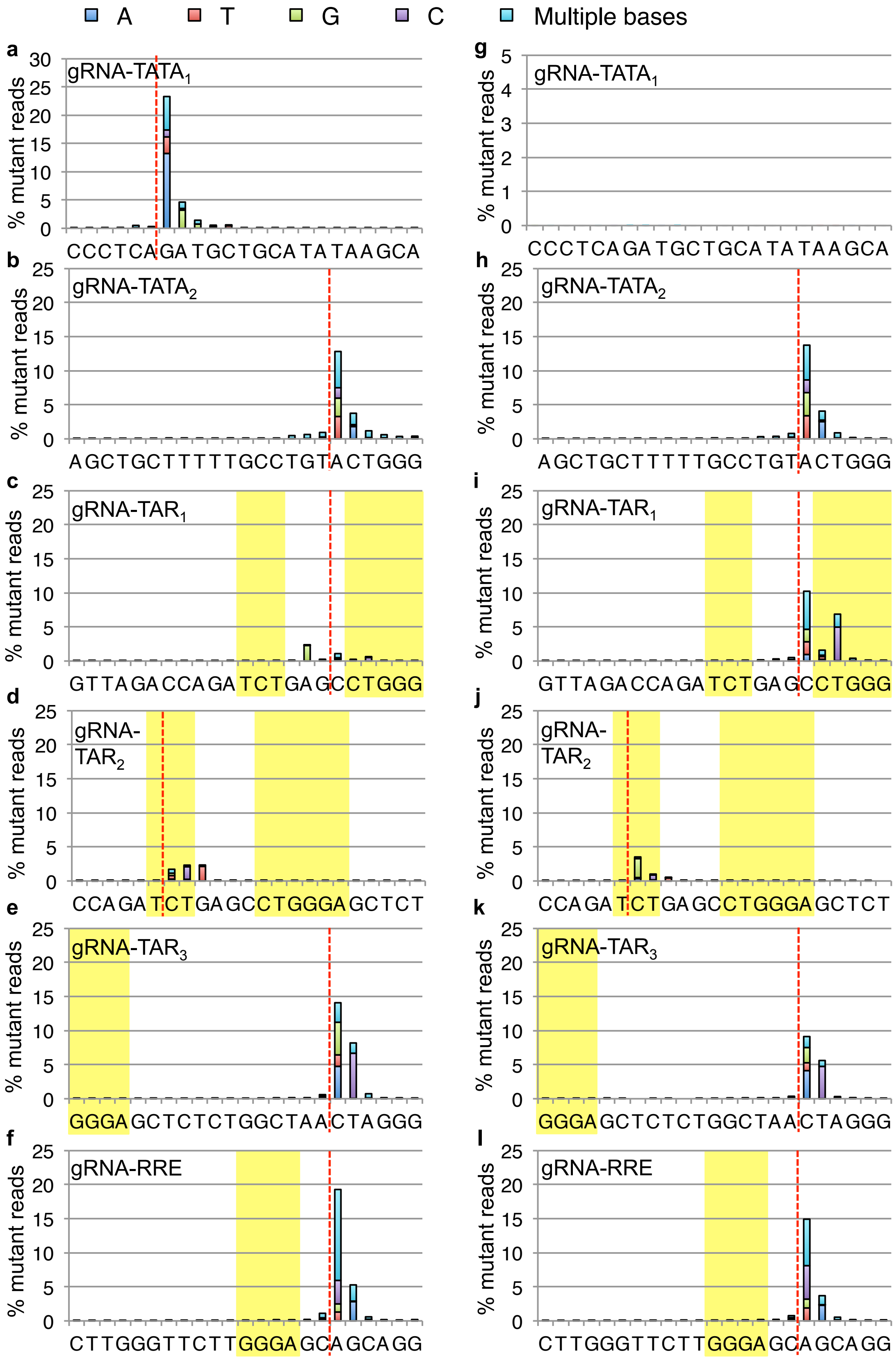

Supplementary Figure 2. Insertion mutations of CRISPR resistance strains do not show preference for any base.

Wild type human CD4+ T cell line SupT1 and derivatives expressing CRISPR Cas9 and multiple gRNAs targeting HIV-1 were infected with strain (**a-f**) NL4-3 or (**g-l**) R7. The 23 bp gRNA targeted sequence is shown. Red dotted lines indicate the site of Cas9 cleavage. Yellow highlighting indicates bases that give rise to a bulge or loop in an RNA structure. Identities of single base insertions as well as multiple base insertions are shown. Insertions are exclusively at the double strand break induced by Cas9.

Supplementary Table 7. Primer sequences

Primer	Sequence	Application
oKEY787	CACCGTGCTTATATGCAGCATCTGA	CRISPR gRNA-1 TATA oligomer
oKEY788	AAACTCAGATgCTGCATATAAGCAC	CRISPR gRNA-1 TATA oligomer
oKEY791	CACCGAGCTGCTTTTTGCCTGTACT	CRISPR gRNA-2 TATA oligomer
oKEY792	AAACAGTACAGGCAAAAAGCAGCTC	CRISPR gRNA-2 TATA oligomer
oKEY795	CACCGGTTAGACCAGATCTGAGCCT	CRISPR gRNA-3 TAR oligomer
oKEY796	AAACAGGCTCAGATCTGGTCTAACC	CRISPR gRNA-3 TAR oligomer
oKEY797	CACCGAGAGCTCCCAGGCTCAGATC	CRISPR gRNA-4 TAR oligomer
oKEY798	AAACGATCTGAGCCTGGGAGCTCTC	CRISPR gRNA-4 TAR oligomer
oKEY793	CACCGCTTGGGTTCTTGGGAGCAGC	CRISPR gRNA-5 RRE oligomer
oKEY794	AAACGCTGCTCCCAAGAACCCAAGC	CRISPR gRNA-5 RRE oligomer
oKEY820	CGCATGTCTGTGGGCTGGGCC	CRISPR gRNA-1 TATA off-target A primer
oKEY861	CCTCCTTCTCTAGCCCTTGCCACATCCAC	CRISPR gRNA-1 TATA off-target A primer
oKEY822	CCTCTGGTCCCAGATGTGTCTC	CRISPR gRNA-1 TATA off-target B primer
oKEY823	GGTTGTGGCGCCCTGCTTTGAC	CRISPR gRNA-1 TATA off-target B primer
oKEY840	AGAATCACAGCACAGTGGAGTACACG	CRISPR gRNA-2 TATA off-target A primer
oKEY825	GGAGTCTGTCGCCCAGGTGGAG	CRISPR gRNA-2 TATA off-target A primer
oKEY826	TAACTCAATCCTCTCAATTTCCAG	CRISPR gRNA-2 TATA off-target B primer
oKEY827	TCGTACCACTTCTCAAACTCCC	CRISPR gRNA-2 TATA off-target B primer
oKEY854	GAGCAGAGTGGCCATACCGGGTTTTCCG	CRISPR gRNA-3 TAR off-target A primer
oKEY855	GGAGTGAGAGTCCGAGGCTCCCATGG	CRISPR gRNA-3 TAR off-target A primer
oKEY830	CCTCCCTTCCCCCCTCTCCAC	CRISPR gRNA-3 TAR off-target B primer
oKEY844	CCTTACTTAGGGTGGATGCTATGCCTCC	CRISPR gRNA-3 TAR off-target B primer
oKEY862	GAGGAAAGATGCCACCCCCTCCCCTGC	CRISPR gRNA-4 TAR off-target A primer
oKEY863	CGCCTAGCAGGGGCAAAGATACAGAGGTAAGG	CRISPR gRNA-4 TAR off-target A primer
oKEY834	ACTTTAACCATGATAGGCATCTC	CRISPR gRNA-4 TAR off-target B primer
oKEY835	GAATTCTGTAACAGATATGCATTTAC	CRISPR gRNA-4 TAR off-target B primer
oKEY847	TGAGCCATGGGGGTGCAGCAGGC	CRISPR gRNA-5 RRE off-target A primer
oKEY856	GGGCATCAGGTCCTCCATCTCACAGTCCC	CRISPR gRNA-5 RRE off-target A primer
oKEY864	CGGACCTGCGACTTCCGAACAACCCTGGC	CRISPR gRNA-5 RRE off-target B primer
oKEY865	CTCTAAGCAGCAAACGAGGGGGCGGAACCTCG	CRISPR gRNA-5 RRE off-target B primer
oKEY764	GGATGGTGCTACAAGCTAGTAC	HIV-1 LTR primer
oKEY782	CTAGAGATTTTCCACACTGACTAAAAG	HIV-1 LTR primer
oKEY217	GCTTGTGTAATTGTTAATTTCTCTGTC	HIV-1 RRE primer
oKEY271	GATATGAGGGACAATTGGAGAAG	HIV-1 RRE primer

Supplementary Table 8. Surveyor off-target sizes

Off-target	Gene	Locus	Amplicon length (bp)	Fragment 1 length (bp)	Fragment 2 length (bp)
gRNA-TATA 1 A	TNK2	chr3:+195605446	486	265	221
gRNA-TATA 1 B	ABHD15	chr17:-27888832	520	254	266
gRNA-TATA 2 A	YTHDC1	chr4:-69177535	629	348	281
gRNA-TATA 2 B	CCDC146	chr7:-76866200	490	214	276
gRNA-TAR 1 A	CELSR1	chr22:-46773194	531	264	267
gRNA-TAR 1 B	SOX2-OT	chr3:+181459426	423	285	138
gRNA-TAR 2 A	LOC146880	chr17:-62778129	610	336	274
gRNA-TAR 2 B	SOX2-OT	chr3:-181459429	545	233	312
gRNA-RRE A	WNT8B	chr21:+44836938	613	301	312
gRNA-RRE B	EPHA1-AS1	chr10:+99497129	526	208	318
